# Alteration of Immune-Mechanisms by Human Microbiota and Development and Prevention of Human Diseases

**DOI:** 10.1155/2017/6985256

**Published:** 2017-12-28

**Authors:** Mitesh Dwivedi, Ansarullah, Ilian Radichev, E. Helen Kemp

**Affiliations:** ^1^C. G. Bhakta Institute of Biotechnology, Tarsadi, Uka Tarsadia University, Surat, Gujarat 394350, India; ^2^Izmir International Biomedicine and Genome Institute (iBG-izmir), Dokuz Eylul University, Izmir, Turkey; ^3^Children's Health Research Center, Sanford Research, 2301 East 60th Street-North, Sioux Falls, SD 57104, USA; ^4^Department of Oncology and Metabolism, University of Sheffield, Sheffield, UK

The role of microbiota in regulating the immune system has gained much importance in recent years, since it plays a crucial role in disease development and prevention. The human body harbors 100 trillion commensal microbes, exceeding the number of host cells by more than 10-fold, with a huge amount of genetic information, that is, 100-fold more genes than the human genome. With this enormous pool of genetic information, the microbiota may influence human life at many levels including host immunity and its regulation. The immune mechanisms elucidating the molecular interactions between the microbiota and host immune system components have come up with new and interesting therapeutic approaches. Recent studies focusing on the interactions between microbiota and the host immune system have revealed the fundamental importance of the microbiome in shaping host immune responses, affecting susceptibility against immune-mediated and infectious diseases.

A total of 15 manuscripts were received for this special issue, and 9 manuscripts have been accepted for publication after rigorous rounds of review process. This issue provides a glimpse of some of the ongoing efforts in the area of immune regulation by microbiota and management of the human health. For example, some featured papers provide useful information regarding the role of microbiota in altered immune mechanisms in human diseases such as rheumatoid arthritis, multiple sclerosis, erythema nodosum, connective tissue diseases and vasculitides, and allergic asthma.

One of the interesting research articles presented by A. Fusco et al. explores the role of *β*-defensins in protecting the intestinal epithelium against *S. typhimurium* and their interaction with the gut microbiota. In particular, the experiments of coinfection with *S. typhimurium* and probiotic *E. faecium* showed that, in the presence of *E. faecium*, *Salmonella* infection caused a much less intense inflammatory response. Overall, the findings of this study propose that antimicrobial peptides (AMPs) could be considered, in the future, a new class of therapeutics since they are able to induce lesser resistance and have a selective antimicrobial activity to protect the host without the need for the immune system memory.

The research article presented by L. M. Rocha-Ramírez et al. reveals the innate immune mechanisms generated due to probiotic *Lactobacillus* strains. In particular, the study demonstrates that strains of *Lactobacillus* exert early immunostimulatory effects that may be directly linked to the initial inflammation of the response of human macrophages. The study further proposes that the effect of these probiotics as potential immunomodulators in immunocompromised hosts could be evaluated.

The research article presented by C. F. Michael et al. provides interesting insights into repair of epithelial damage in asthmatic airways by using prebiotic mannan (SC-MN) derived from *S. cerevisiae*. Unlike the mannan derived from bacteria, SC-MN does not stimulate Th17 inflammatory cytokines and the study reports that SC-MN showed an increased expression and activation of Krüppel-like factors (KLFs) 4 and 5, key transcription factors for epithelial cell differentiation, survival, and proliferation. Since anti-inflammatory therapy alone has not been shown to halt disease progression in asthma, the study proposes a therapy that could promote epithelial repair and provide adjunctive therapeutic benefit for asthma. In this line, another research article by D. B. Lew et al. demonstrated the beneficial effects of SC-MN on allergic asthma mouse models. In particular, the study showed that the antismooth muscle (ASM) hyperplasia/hypertrophy in the mouse model due to allergen challenge can be preventable with SC-MN treatment. The study further concludes that the prebiotic mannose receptor blocker SC-MN is a promising agent that can render dual benefits in asthma, that is, anti-inflammatory and ASM remodeling at the level of both large and small airways. Another research article by O. Ozbagcivan et al. presents a retrospective descriptive study which examines the frequency and type of infections in the etiology of erythema nodosum (EN). The study identified, besides *Streptococci*, many other microbes including the ones living on and inside the human body to be of importance in the etiology of EN.

The current issue also involves interesting review articles. G. Horta-Baas et al. present a review article which provides some evidence linking intestinal dysbiosis with the autoimmune mechanisms involved in the development of rheumatoid arthritis (RA). The article reviewed various studies that have evaluated the influence of gut microflora on the etiopathogenesis of RA as well as RA severity. The article proposes that a specific or systematic manipulation of certain intestinal microbiota associated with host diseases could change therapeutic strategies in subjects with RA.

Further, G. Ranucci et al. present a review article on the role of early life intestine microbiota in the lungs' health in children. In particular, the review article discusses the recent development in gut-lung axis research, with emphasis on the effects of targeting microbiota of infants and children at a risk of or with progressive lung diseases. The article proposes that such knowledge could open new possibilities for therapeutic interventions in modifying the progression of chronic lung diseases as well as preventing its onset in population at risk.

The recent advances in sequencing technologies allowed for an unprecedented growth in the information about the diversity of bacterial species providing a valuable information how this diversity is associated with human health and disease. In this special issue, R. Talotta et al. review the latest information about the role of human microbiota in the pathogenesis of connective tissue diseases (CTDs) and vasculitides. This is a highly understudied field, and the authors discuss the potential links between dysbiosis and pathogenesis of inflammatory arthritides, such as systemic lupus erythematosus, systemic sclerosis, Sjӧgren's syndrome, and Behçet's disease. A more targeted evaluation of the impact of gut microflora on the pathophysiology of multiple sclerosis (MS) has been discussed by M. Adamczyk-Sowa et al. The results showed that the MS patients' intestinal microflora is characterized by moderate dysbiosis in combination with the protective effect of some *Bacteroides* metabolites against demyelinating in animal models, which suggests the existence of a very close interaction between the intestinal mucosa and the brain.

Thus, many previous and current reports suggest that there is a captivating relationship between microbiota and immune system. Although a few mechanisms of immune regulation by microbiota have been described, the exact role of microbiota in various diseases is still unknown. However, interventional studies are needed to elucidate such functional aspects of microbiota or their product's role in immune regulatory mechanisms. This knowledge will be of importance in order to optimize clinical studies and gain deeper mechanistic insights.

In summary, this special issue covers many important aspects of microbiota role in immune regulation and human health. We hope that this special issue can provide valuable information to investigators in the field of microbiota and immune regulation and also give the readers a sense of the advancements made in this field.

